# Efficacy of Platelet-Rich Fibrin Combined with Autogenous Bone Graft in the Quality and Quantity of Maxillary Alveolar Cleft Reconstruction

**Published:** 2018-11

**Authors:** Maryam Omidkhoda, Arezoo Jahnabin, Fatemeh Khoshandam, Farzaneh Eslami, Seyed Hossein Hosseini Zarch, Jalil Tavakol Afshari, Hamed Kermani

**Affiliations:** 1 *Department of Orthodontics, School of Dentistry, Mashhad University of Medical Sciences, Mashhad, Iran.*; 2 *Dentist, Private Practice, Mashhad, Iran.*; 3 *Department of Oral and Maxillofacial Radiology, School of Dentistry, Mashhad University of Medical Sciences, Mashhad, Iran.*; 4 *Immunology Research Center, Department of Allergy and Immunology, School of Medicine,, Mashhad University of Medical Sciences, Mashhad, Iran.*; 5 *Department of Oral and Maxillofacial Surgery, School of Dentistry, Tehran University of Medical Sciences, Tehran, Iran.*

**Keywords:** Alveolar graft, Cleft lip and palate, Platelet-rich fibrin

## Abstract

**Introduction:**

The aim of this study was to evaluate the effect of platelet-rich fibrin (PRF) on the quality and quantity of bone formation in unilateral maxillary alveolar cleft reconstruction using cone beam computed tomography.

**Materials and Methods:**

This study was conducted on 10 non-syndromic patients with unilateral cleft lip and palate within the age group of 9-12 years. The study population was randomly assigned into two groups of PRF and control, each of which entailed 5 cases. In the PRF group, the autogenous anterior iliac crest bone graft was used in combination with PRF gel. On the other hand, the control group was subjected to reconstruction only by bone graft. The dental cone beam CT images were obtained immediately (T0) and 3 months (T1) after the operation to assess the quality and quantity of the graft. Independent and paired sample t-tests and analysis of covariance were used to analyze and compare the data related to the height, thickness, and density of the new bone.

**Results:**

The mean thickness difference of the graft in both PRF and control groups at T0 and T1 was not significantly different (P>0.05). Furthermore, the reduction changes of bone height at the graft site from T0 to T1 were not statistically significant for both groups (P=0.78). The mean total bone loss of the regenerated bone from T0 to T1 was lower in the control group than that in the PRF group; however, this difference was not statistically significant.

**Conclusion:**

The usage of PRF exerted no significant effect on the thickness, height, and density of maxillary alveolar graft.

## Introduction

Cleft lip and/or palate is the most common congenital anomaly that affects the orofacial region. Accordingly, significant efforts have been made to manage these anomalies. Patients suffering from this condition usually need different surgical interventions; however, there is no standard protocol for the treatment of this anomaly. Repair of the alveolar cleft with bone grafting is a necessary adjunct procedure that is recommended during the mixed dentition period (1). 

Bone grafting is used to improve function and esthetics for patients with unilateral or bilateral cleft lips and palates by the improvement of oral hygiene, stabilization of the maxillary arch, closure of the oral fistula, normalization of growth at the cleft site, and creation of bony support for the eruption of adjacent permanent teeth (2,3). Autogenous bone is currently preferred among the different graft materials available for the reconstruction of the cleft site (4). The sources of autogenous bone include grafting from the anterior iliac crest, ribs, symphysis, and tibia (5). According to the literature, the bone graft harvested from the anterior iliac crest is considered as the gold standard source for the reconstruction of alveolar clefts (6-8). Some recent studies (9-11) have shown that osteoinductive or osteoconductive growth factors, such as platelet products like platelet-rich fibrin (PRF), significantly improve the bone repair. The PRF is a new generation of platelet concentrate that is simple to prepare without the need for anticoagulant or other artificial biochemical modifications. This biomaterial is prepared from patient’s own blood and (6,9). It contains platelet-derived growth factor, vascular endothelial growth factor, and modified transforming growth factor ß1 (12). The PRF accelerates the regeneration and healing of the wound (9,13). 

With this background in mind, the present study was conducted to compare the efficacy of autogenous bone graft and the combination of PRF with autogenous bone graft in the quantity and quality of the newly formed bone after the reconstruction of maxillary alveolar cleft.

## Materials and Methods

Ethical approval for this clinical trial was granted by the Medical Ethical Committee and the Research Deputy of Mashhad University of Medical Sciences. This study was conducted on 10 non-syndromic patients with unilateral cleft lip and palate (i.e., 4 females, 6 males) within the age range of 9-12 years (mean age: 11.3±0.83 years), referred to the Cleft Lip and Palate Center at Mashhad School of Dentistry. 

The inclusion criteria were: 1) unilateral cleft lip and palate needing maxillary expansion before alveolar bone grafting,2) no systemic disease, 3) good oral hygiene, 4) no previous grafting attempts at the cleft site, 5) no local problem in the maxilla that could interfere with surgery, and 6) parental informed consent. On the other hand, the exclusion criteria included: 1) unwillingness to participate in the study, 2) special systemic disease, and 3) no need for maxillary expansion before surgery.All patients were subjected to a thorough preoperative examination, including a medical history taking and a physical examination by a cardiologist to exclude any systematic disease that might interfere with the operation process. At this step, the patients were randomly divided into two groups of PRF (n=5) and control (n=5).

*Preparation of platelet-rich fibrin*

**Fig1:**
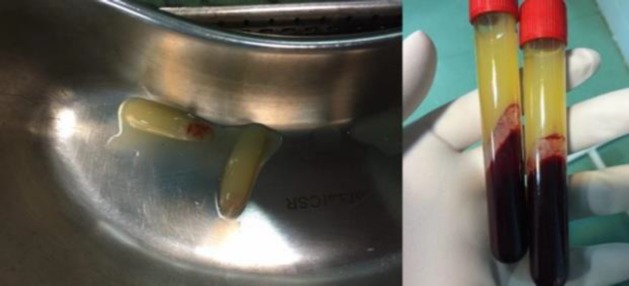
Preparation of PRF gel

 Prior to the surgery, 20 ml fresh venous blood was taken from each patient and transferred into sterile tubes. As a standard protocol, the tubes were then quickly placed into the Pc-02 table centrifuge (Process, Nice, France), which was adjusted to 3,000 rpm for 10 min (9,11). The tubes were then removed from the centrifuge. Given the lack of anticoagulant in the tubes, there were three distinct layers inside each tube. These layers included platelet-poor plasma at the topmost layer, PRF in the middle zone, and the red blood cells in the lowest layer (9,14) (Fig.1).

*Operative procedure*

 Surgery was performed after the retention period of maxillary expansion with the W-arch kept in place. Alveolar bone grafting was carried out under general endotracheal anesthesia. A gingival mucoperiosteal flap was created for better access to the surgical site. Autogenous cancellous bone and marrow from the anterior iliac crest were used for each patient. The PRF was mixed with the bone graft to obtain a substance with a gel-like consistency. This mixture was packed into the alveolar cleft site in the PRF group, and then the flap was closed. The patients were prescribed adequate post-surgical anti-inflammatory medication and antibiotics. The surgeon followed up the patients for two weeks after the surgery.

*Radiographic evaluation*

The evaluation of the quality and quantity of the graft in the cleft area was accomplished using the cone beam CT (CBCT) images. The images were taken at two stages, namely immediately after the operation (T0) and 3 months later (T1), using a ProMax three-dimensional model CBCT (Planmeca, Finland, 2009). The exposure parameters included a field of view of 90×100 mm, voxel size of 200 μm, X-ray tube kilovoltage of 88 kVp, and 8 mA. An oral and maxillofacial radiologist evaluated and superimposed the images using the Romexis software package (version 3.4.4).

At the first step, the CBCT images of T0 and T1 were superimposed in common sites with the least changes over time, such as the skull base and orbit in coronal, axial, and sagittal planes. After the superimposition of the midsagittal planes of two CBCTs by the operator, the software automatically fitted them to make the best superimposition.

**Fig 2:**
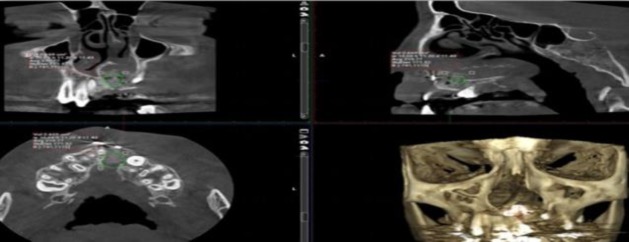
Measuring density of the graft

The bone density was evaluated using the Hansfield unit (HU) in a qualitative analysis. In order to evaluate the bone density of the graft, common graft areas in both images of each patient were diagnosed. As the grafted bone was fully distinguished from the adjacent bones, a circle was drawn by the software in equal dimensions just in the graft area for the purpose of density measurement (Fig.2).

**Fig 3:**
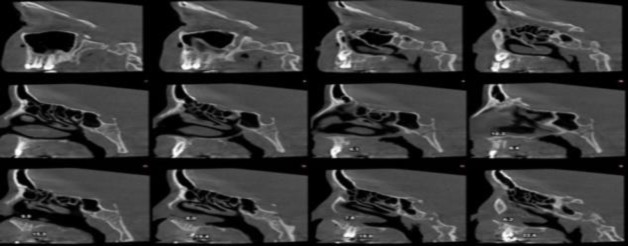
Evaluating the height and the thickness of the bone graft

As the grafted bone was completely recognizable from the surrounded bones, in each image, the height of the graft (H) and its thickness were measured in all CBCT cuts in millimeters, and their mean values were used for statistical analysis (Fig.3).

All measurements were performed and double checked by an oral and maxillofacial radiologist.

*Statistical analysis*

Statistical tests were conducted in PASW^®^, version 18 (SPSS; Chicago, Illinois, USA). One-sample Kolmogorov-Smirnov test was used to evaluate the normality of the data. Furthermore, independent sample t-test, paired t-test, and analysis of covariance were employed to analyze and compare the data related to the graft height, thickness, and density. P-value less than 0.05 was considered statistically significant.

## Results

The Kolmogorov-Smirnov test showed that all data were normally distributed (P>0.05). The evaluation of the changes in graft thickness from T0 to T1 was accomplished using the independent sample t-test. The results showed that these changes were not statistically significant in both groups (P=0.66). There was no significant difference between the PRF and control groups in terms of the mean thickness difference of the graft at both T0 (P=0.92) and T1 (P=0.8). 

Based on the results of the paired sample t-test, the PRF group showed statistically significant changes in the graft thickness at T1, compared to that at T0 (P=0.007; Table.1). The analysis of covariance showed that the bone thickness reduction of the graft packed in the cleft area in the PRF group was 0.9 mm more than that in the control group after 3 months, which was not statistically significant (P=0.69).

**Table 1:** Mean values of graft thickness in case and control groups immediately and three months after operation

**Group**	**T0**	**T1**	**T1-T0**	**Paired sample t-test results**
	**Mean±SD**	**Mean±SD**	**Mean±SD**	
Control	13.9±3.7	10.7±4.7	3.1±1.9	t=1.7 P=0.16
PRF	14.1±2.1	10.0±3.9	2.8±1.7	t=5.0 P=0.007
Independent sample t-test	t=0.09 P=0.92	t=0.25 P=0.80	t=0.44 P=0.66	

T0: Immediately after the operation, T1: Three months after the operation, PRF: platelet-rich fibrin

According to Table 2, there was no statistically significant difference between the two groups in terms of the mean graft height at both T0 and T1 (P>0.05). Therefore, the reduction changes at the graft site from T0 to T1 were not statistically significant for both groups (P=0.78). Based on the covariance analysis, although graft height reduction in the PRF group was 0.4 mm lower than that in the control group at T1, the results failed to show a significant difference between the two groups in this regard (P=0.69). 

**Table 2:** Mean values of graft height in case and control groups immediately and three months after operation (mm)

**Group**	**T0**	**T1**	**T1-T0**	**Paired t- test results**
	**Mean±SD**	**Mean±SD**	**Mean±SD**	
Control	14.0±4.0	10.9±3.6	3.1±1.9	t=3.6 P=0.02
PRF	11.3±5.1	8.5±3.6	2.8±1.7	t=3.5 P=0.02
Independent sample-t-test	t=0.92 P=0.38	t=1.03 P=0.33	t=0.28 P=0.78	

T0: Immediately after the operation, T1: Three months after the operation, PRF: platelet-rich fibrin

In the PRF group, the mean bone densities of the graft were 404.1±170.9 and 302.83±128.82 HUs at T0 and T1, respectively. These mean values were obtained as 438.3±135.63 and 349.6±172.6 in the control group, respectively. Based on the results, the mean total bone loss of the graft from T0 to T1 was lower in the control group than that in the case group; nonetheless, it was not statistically significant (P=0.83; Table.3).

**Table 3:** Mean values of graft density in case and control groups immediately and three months after operation (mm)

**Group**	**T0**	**T1**	**T1-T0**	**Paired sample ** **t- test results**
	**Mean±SD**	**Mean±SD**	**Mean±SD**	
Control	438.26±135.63	349.58±172.58	88.67±82.48	t=2.40 P=0.07
PRF	404.09±170.88	302.83±128.82	101.26±96.10	t=2.35 P=0.07
Independent sample t-test	t=0.35 P=0.73	t=0.48 P=0.64	t=0.22 P=0.83	

T0: Immediately after the operation, T1: Three months after the operation, PRF: platelet-rich fibrin

## Discussion

Closure of the alveolar cleft is an important stage in the treatment of patients with cleft lip and palate. This practice may have some potential benefits, such as facilitating orthodontic treatment and tooth replacement in the cleft area. The use of platelet products, such as PRF, is a new, promising approach in the field of dentistry, especially for clinical conditions requiring rapid healing in both soft and hard tissue (13).

The aim of the present study was to reconstruct the maxillary alveolar clefts using the combination of PRF and autogenous bone graft. Half of the patients were subjected to this reconstruction using the PRF gel in combination with bone graft, while the other half were managed only by means of the bone graft. The results of our study demonstrated that the use of PRF gel with the autogenous bone graft in the cleft site exerted no significantly different effect on the quality and quantity of the graft in the cleft area 3 months after the operation.

Anwandter et al. and Wang et al. studied the alveolar ridge dimensional changes after tooth extraction (15,16), which was preserved with PRF and stated that PRF could not preserve the alveolar socket from vertical and horizontal bone loss. In another study performed by Gurler et al. on the effect of PRF combined with bone graft on sinus lifting, it was reported that the use of PRF did not improve the situation significantly (17). 

In the abovementioned studies, the situation of the bone defect was totally different from that investigated in our study, in which grafts were used to close the cleft site. Nevertheless, it can be concluded from these studies that the use of PRF as a regenerative substance is not beneficial.

One study (14) indicated the significant positive stimulatory effect of PRF in repairing alveolar bone defects post-operation on impacted bilateral third molars. However, in the mentioned study, periapical radiographs were used to evaluate the density of the bone in that area, which might not be sufficiently accurate for the assessment of bone density in the area. On the other hand, in the present study, CBCT imaging was employed, which allows for the accurate evaluation of bone graft changes.

In an animal study, Yuanzheng et al. (18) used PRF or mesenchymal stem cells with the iliac graft in 20 dogs with unilateral alveolar clefts. They stated that the sole or combined use of PRF with stem cells could be effective in maintaining the volume and density of the alveolar graft bone 6 months after the surgery. The difference between the results of the present study and the mentioned research could be due to the difference in sample size, measurement protocols, post-surgery follow-up period, operation technique, and nature of working on animal participants.

Seifeldin and Shawky (19) performed a similar study on 24 patients with unilateral alveolar cleft, using PRF with iliac bone graft to reconstruct the alveolar bone. The CT images were obtained immediately post-surgically and 6 months later. They reported that the use of PRF in combination with the autogenous iliac bone graft was beneficial in the improvement of bone volume (i.e., quantity) in reconstructing the cleft area; nonetheless, it failed to enhance the bone density (i.e., quality). Given the conflicting results described in the limited literature related to the efficacy of PRF in the alveolar bone grafting, it is suggested to perform further in vivo studies with a larger sample size and longer follow-up periods to clarify the actual advantages of PRF in patients with cleft lip and palate.

## Conclusion

As the findings of the present study indicted, the application of PRF in combination with autogenous bone did not have any significant effect on the thickness, height, and density of maxillary alveolar graft in a three-month period.
